# Liquid lithium as divertor material to mitigate severe damage of nearby components during plasma transients

**DOI:** 10.1038/s41598-022-21866-1

**Published:** 2022-11-05

**Authors:** V. Sizyuk, A. Hassanein

**Affiliations:** Center for Materials Under Extreme Environment (CMUXE), Purdue University, West Lafayette, IN 47907 USA

**Keywords:** Physics, Plasma physics, Magnetically confined plasmas

## Abstract

The successful operation of thermonuclear fusion reactors such as ITER, DEMO, and future commercial plants is mainly determined by the optimum choice of materials for various components. The objective of this work is to accurately and comprehensively simulate the entire device in 3D to predict pros and cons of various materials, e.g., liquid lithium in comparison to tungsten and carbon to predict future ITER-like and DEMO divertor performances. We used our comprehensive HEIGHTS simulation package to investigate ITER-like components response during transient events in exact 3D geometry. Starting from the lost hot core plasma particles through SOL, deposition on the divertor surface, and the generation of secondary plasma of divertor materials. Our simulations predicted significant reduction in the heat loading and damage to the divertor nearby and internal components in the case when lithium is used on the divertor plates. While if tungsten or carbon are used on the divertor plate, significant melting areas and vaporization spots can occur (less for carbon) on the reflector, dome, and stainless steel tubes, and even parts of the first walls can melt due to the high radiation power of the secondary divertor plasma. Lithium photon radiation deposition into the divertor and nearby surfaces was decreased by two orders of magnitude compared to tungsten and by one order of magnitude compared to carbon. This analysis showed that using liquid lithium for ITER-like surfaces and future DEMO can lead to significant enhancement in components lifetime.

## Introduction

The successful development of thermonuclear fusion reactors such as ITER or next generation DEMO devices is mainly determined by the optimum choice of materials for the various components and systems. The material selections should promote long lifetime of the components (especially divertor) including tolerance to the high heat loads during plasma transient events, provide efficient thermonuclear reaction and energy transformation, retain minimum tritium concentration in components, promote material compatibility issues, safety and other requirements. Currently, ITER is the main international project aiming to demonstrate the capability of the tokamak concept for future energy production. ITER device is a much larger than any existing current tokamaks and will have much higher heat fluxes to the divertor components during plasma instabilities. The expected surface heat loads during plasma material interaction (PMI) is one of the main limitations in the development of successful fusion devices. The plasma facing components (PFCs) will be damaged and eroded in the ITER device not only during abnormal operation (e.g., disruption) but also at normal operation, i.e., edge-localized modes (ELMs)^1^. Using full tungsten divertor as in the current ITER design could cause significant damage to all interior components not initially visible to the disrupting plasma including baffles, reflector plates, dome, and even the beryllium first wall. To repair all of these components will require significant downtime in reactor operation for extended periods. The full tungsten design of ITER divertor during plasma instabilities will result in development of dense high-Z secondary tungsten plasma with very high radiation power to various interior components.

One proposed way to decrease interior components heat loading is to partially cover or insert strip of low-Z materials around the strike points (SP) of the tungsten divertor. Small carbon inserts at the SP, for example, can eliminate or significantly reduce tungsten content in the secondary plasma, i.e., carbon generated plasma, reducing core plasma tungsten contamination and greatly decrease the damage of the divertor nearby surfaces and first walls due to the much reduced radiation power^2^. A small strip of carbon insert (only less than 10% of the all carbon divertor plate design option, which has its own additional problems) will prevent the damage of all these interior components that are very hard to repair and will prevent the potential for significant amount of high-Z contamination to the core plasma during transient events which can then cause full disruption or affects the successful operation in the current ITER design. The carbon generated plasma absorbs energy mainly into the thermal part in comparison to the high-Z tungsten. Carbon has a simple atomic structure compared to tungsten. As a result, tungsten ions consume much of the transient plasma energy through ionization while in carbon is by increasing their ions velocity. The advantage of using carbon is that the thermal cooling is slow process. The final energy deposition will be delayed in time and localized within carbon particles which is transferred to far locations with very low intensity that do not cause significant damage. In the tungsten case, cooling process is the recombination of W ions and strong photons emission. This process is much faster and the final energy deposition is not localized within tungsten ions due to the reradiated photons moving in all directions regardless of the magnetic field structure. Because the tungsten ions are heavier than the carbon ions, the collisional and scatterings processes are more "effective" in the tungsten case, i.e., more of the incident hydrogen ions and their energy change direction and are reflected to walls and internal components and do not penetrate deeply into the secondary dense plasma cloud. As result, the final energy deposition is redistributed to interior component surfaces causing intense local hot spots.

However, using carbon as PFC has also several disadvantages including higher erosion, tritium retention issue, dust in chamber, severe neutron damage, etc. There are methods previously proposed to remove tritium from carbon and CFC, e.g., heating in between discharges using laser beams, etc. In fact, transient events themselves like ELMs and disruptions on the small strip of carbon will actually help to remove tritium due to the high temperatures during these events. Most of the interior design, e.g., dome, baffles, reflector plates, and most of the divertor plate are still made of tungsten. The thin carbon insert is a compromise between the full tungsten divertor and divertor with entire carbon plate, which is not currently favored. Both options have advantages and disadvantages. The installation of a very small and easily replaceable low-Z carbon insert on the tungsten plate can significantly protect all nearby surfaces and first wall from serious damage and can extend the lifetime of divertor components^2^.

The next generation DEMO fusion power plant is planned to be a device between ITER and a commercial fusion power plant^3^. This DEMO should demonstrate stable long term operation with net electricity production of few hundred MWs. The divertor and other plasma-facing surfaces will be exposed to much higher energy fluxes in comparison to ITER. The DEMO project is proposed to use liquid lithium as PFC instead tungsten, operate at no-ELM regimes, and avoid or mitigate disruptions. Liquid lithium is capable to solve not only PFCs erosion problem but also be effective heat transporter, tritium breeding material, and enhance performance of the core plasma. These incontestable advantages of liquid lithium allowed to consider it as construction material at a certain stage of ITER project^4^. The lithium self-cooling blanket is the main concept for the DEMO that was planned to be tested during ITER project^5^.

The objective of this work is to accurately and comprehensively simulate the advantages of the liquid lithium material in ITER-like design and conditions and to compare with tungsten and carbon to assess the DEMO performance. We simulated the response of ITER components during plasma transient events starting from the escaping hot core plasma particles all the way down to the generation of secondary divertor plasma and the interaction with various surrounding PFCs.

### Integrated model components

**Figure 1:**
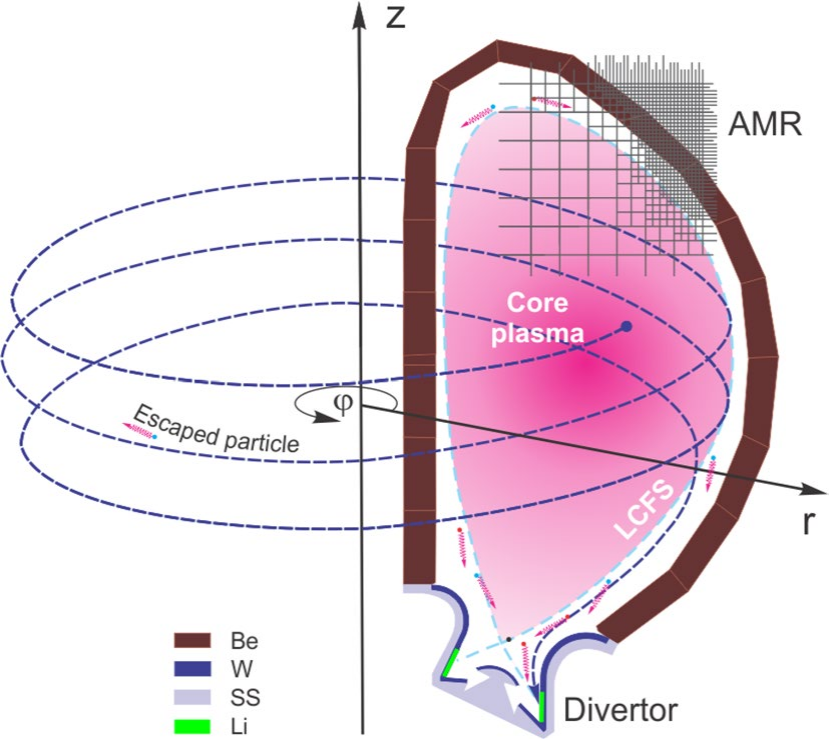
3D schematic illustration of ITER components and coordinate system. The images were prepared using CorelDRAW Graphics Suite 11.

We have enhanced our HEIGHTS full 3D integrated simulation package for lithium calculations including detail photon radiation transport (RT) and focused the present study to investigate the heat loads and damage to various PFCs surfaces during the transient events of ELMs and disruptions^6^. As in our previous studies, we assumed 1 ms duration of these events for the current ITER design^7–9^. Figure 1 schematically shows the three-dimensional computation domain and the coordinate system used. The adaptive mesh refinement (AMR) is used for accurate description of the exact original 3D ITER design geometry from sub-micron to meters long^10^.

**Figure 2:**
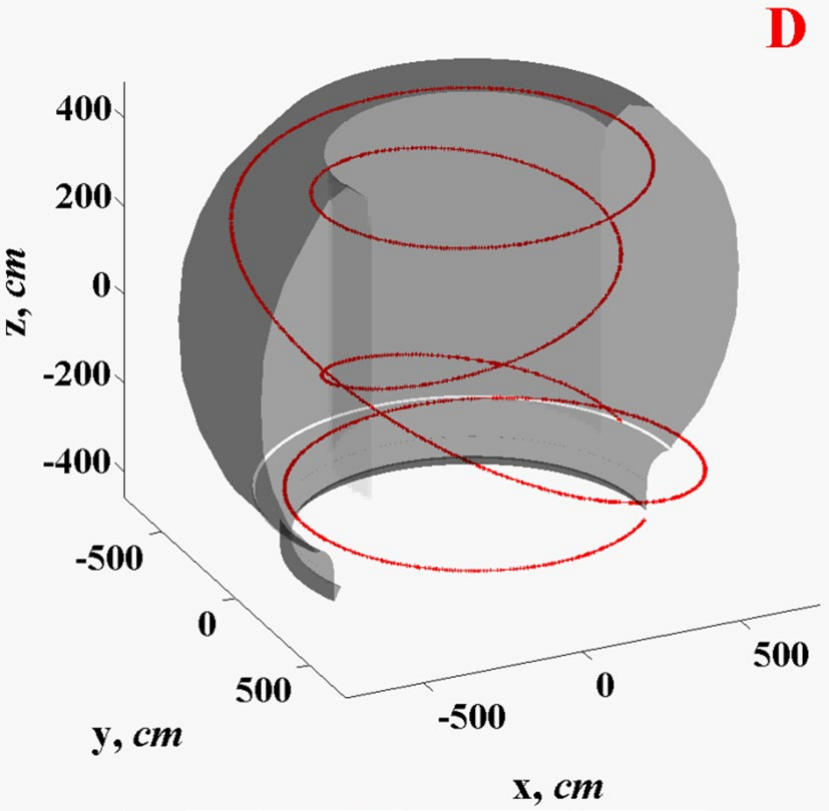
HEIGHTS simulated trajectory of the core escaped deuterium ion in ITER SOL. (See Supplementary Video S1).

The quad-three AMR has 5 layers with minimal MHD cell size ~ 5 mm. The escaped hot plasma core particles gyrate in the toroidal direction starting from the last closed flux surface (LCFS) up to the impact into PFCs surfaces. The most probable impact area at the beginning of transient event is the SP on divertor plates where the lithium trays are installed (Fig. 1, green). On the first stage of our simulation, the evolutions of the escaped particles are used for calculation of the actual energy deposition into the tokamak surfaces and the divertor vapor/plasma evolution and propagation in SOL. We developed gyrokinetic Monte Carlo models for the description of core plasma energy transport^6,11^. Within the frames of our models, the particles gyration is calculating in full 3D (not in so called guiding center approximation^12^) to accurately taking into account the angular changes during the scattering processes. We included into the scattering models eight main physical processes (in the SOL and below the surface): ion-nuclear interactions, ion–electron interaction, electron-nuclear interaction, electron–electron interaction, Bremsstrahlung process, Compton processes, photo-absorption, and Auger recombination^6^. Figure 2 shows sample of the simulated trajectory for the escaped deuterium ion in the SOL, (see Supplementary Video S1 for the simulated dynamics of the escaped from the core region electron and hydrogen ions). The gyrokinetic model describes the rarefied hot core plasma, while the MHD model simulates evolution of the dense secondary plasma initiated after divertor vaporization. The secondary plasma (Li in this case) is several orders denser than the rare core plasma and the MHD treatment is justified for the dense plasma^13^. Our simulations predicted density of secondary plasma up to ~ 10^17^ cm^−3^ in comparison to ~ 10^13^ cm^−3^ for the hydrogen plasma. The gyrokinetic model dynamically recalculates the core plasma flux and energy deposition every several MHD time steps and to every area/component inside the tokamak chamber. The escaped core particles energy (1) deposits into and heat the evolving dense secondary plasma which (2) moves the frozen magnetic field lines that (3) determine the escaped incoming plasma particles trajectories. More details of this self-consistent full 3D scheme can be found in Ref.^2^.

In spite of the lithium being a low-Z material and has much simpler atomic structure than tungsten for example, we did not ignore any of the details of the atomic physics and photon radiation transport (RT) calculations in the lithium secondary plasma. The RT calculations were performed taking into account more than ~ 2800 spectral groups in the range from 0.05 to 10^5^ eV (full spectrum). The details of the RT physics and models in HEIGHTS are presented in Refs.^2,14^. The plasma heat conduction and magnetic diffusion models^15^, the bulk material heat conduction and vaporization models^16^ complete the HEIGHTS self-consistent integrated models.

### Simulation results

**Figure 3:**
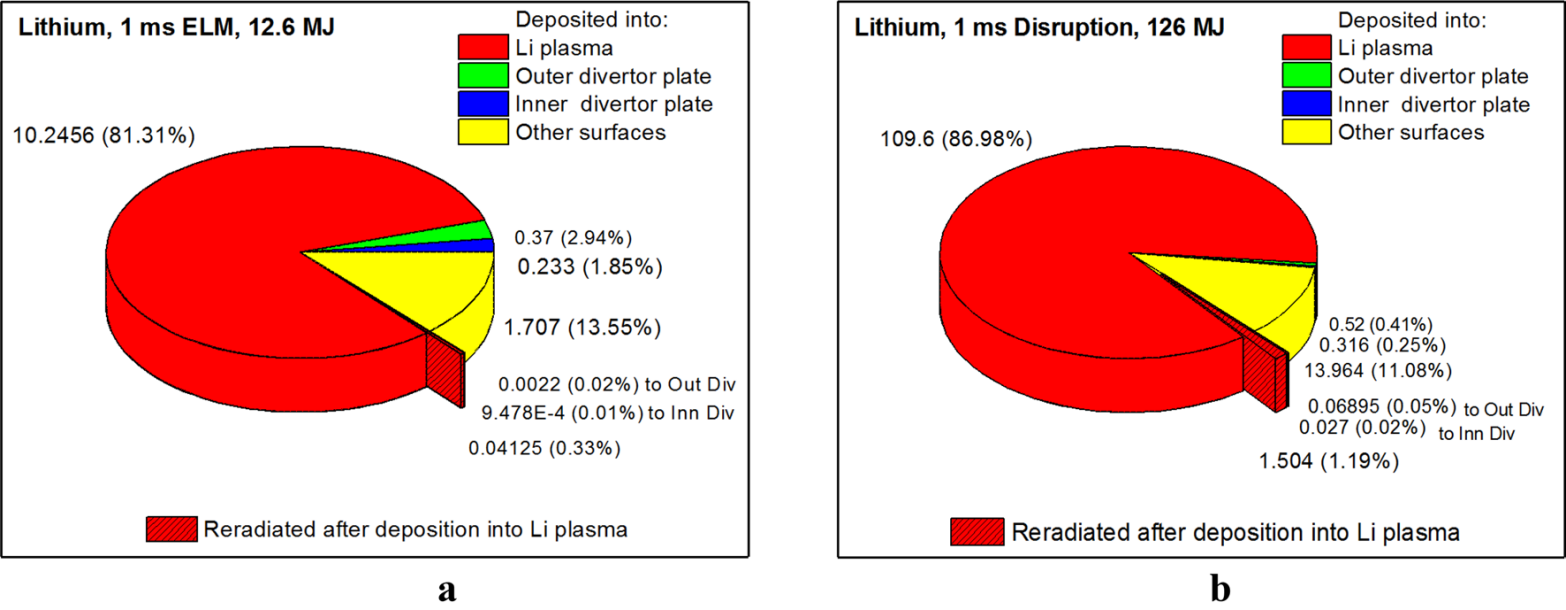
HEIGHTS predictions of the final energy balance in ITER transient events with lithium: 1.0 ms ELM (**a**); and 1.0 ms disruption (**b**). The images were prepared using OriginPro V2020.

In our numerical study, we assumed for the 1 ms disruption the release of full pedestal energy Q_DIS_ = 126 MJ and for the 1 ms giant ELM only 10% of pedestal energy (Q_ELM_ = 12.5 MJ)^2^. The pedestal plasma temperature was taken T_ped_ = 3.5 keV. Based on the transient event total energy, we expressed the final energy distribution balance to all major PFCs in percentages for ITER ELM and disruption (Fig. 3). The escaped particle energy deposited into the Li plasma is marked red, the outer divertor plate green, the inner divertor plate blue, all other surfaces yellow. The analysis of the energy redistribution in the lithium case is compared with tungsten and carbon cases^2^.

As we reported earlier^2^, the low-Z carbon plasma has much lower photon radiation power due to its atomic structure; opposite to the high-Z tungsten plasma. Part of the total ELM energy (12.6 MJ) deposited into the carbon plasma increased up to 10.2 MJ compared to 8.6 MJ for tungsten. In addition, carbon plasma only reradiated 0.62 MJ in photon energy compared to 6.47 MJ for tungsten plasma. Photon radiation is highly difficult to mitigate and its transfer time is very short in comparison to the transport of the thermal plasma energy and is not affected by the magnetic field structure. The present simulations of Li as potential divertor material showed further decrease in photon radiation power compared to carbon.

We summarized the results of the energy distribution for W, C, and Li divertor in ITER design in Table 1. As shown, the total energy deposition into Li secondary plasma is similar to carbon plasma case but the direct core plasma deposition into the divertor plates is much smaller (about three times lower) in the Li case. This can be explained by the easily vaporized Li material with fast formation of plasma cloud and divertor plates shielding. As we predicted above, the Li secondary plasma is much less radiative even in comparison with the low-Z carbon plasma. During the ELM, the tungsten plasma reradiates 51.34% of energy, the carbon plasma 4.92% of energy, and the lithium plasma only 0.36%. The expected photon radiation deposition and damages of surfaces are very small in the Li case. The radiation energy deposition back to the divertor plates is ~ 0.01–0.05% of the total impact energy. The core plasma energy is mainly converted into thermal energy of the secondary plasma in the Li case. The thermal energy transport is much slower compared to the fast transport of the radiation energy where the transport velocity is determined by the speed of light. Our simulations showed that the poloidal velocity of the secondary plasma is in the order of several hundred meters per second. As a result, the heat load on the divertor components is spread in time that allows for such heating mitigation.

**Table 1:** Final energy balance in ITER transient events with W, C and Li strike points.

	Deposited with escaped particles			Reradiated with photons		
	into sec. plasma	into outer plate	into inner plate	from sec. plasma	into outer plate	into inner plate
	ELM/DIS, %	ELM/DIS, %	ELM/DIS, %	ELM/DIS, %	ELM/DIS, %	ELM/DIS, %
W	68.2/85.1	11.11/1.29	7.62/1.02	51.34/44.27	3.81/1.39	1.90/1.18
C	81.0/92.2	6.27/1.33	3.73/0.79	4.92/4.95	0.19/0.13	0.13/0.08
Li	81.7/88.2	2.94/0.41	1.85/0.25	0.36/1.26	0.02/0.05	0.01/0.02

**Figure 4:**
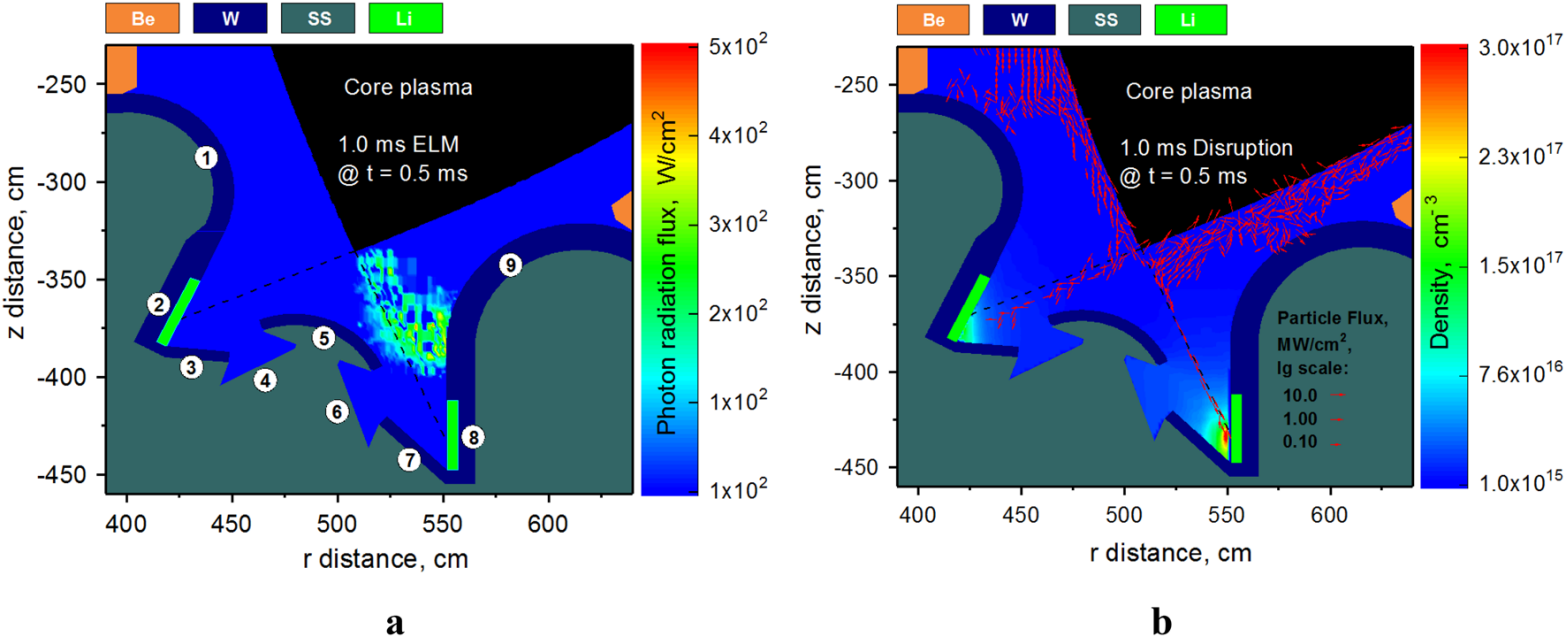
Snapshot of calculated fluxes of Li secondary plasma at t = 0.5 ms: (**a**) photon radiation flux during 1.0 ms ELM (**a**); core plasma particles flux (vectors to scale) during 1.0 ms disruption on background of Li plasma atomic density (**b**). The images were prepared using OriginPro V2020.

The Table 1 reflects the total integrated values in time. However, the transient events in tokamaks have complex self-consistent character with probabilistic distribution in time and space. We should highlight here two main damage sources: the scattered core plasma particles and the photon radiation from the dynamically evolving secondary plasma propagating through the SOL. The time integrated radiation energy shows minimum risk for damage to PFC surfaces. The plotted radiation field in the divertor space (Fig. 4a) shows the two orders of magnitude smaller photon radiation flux in the Li case compared to the W and C secondary plasma (see Fig. 6 of Ref.^2^,). All three cases are plotted at the time moment of 0.5 ms during the 1.0 ms ELM.

As in our previous simulations, we followed the same numeration of component surfaces where #1, #9 are Baffles; #2, #8 are Divertor Plates; #3, #7 are Reflectors; #4, #6 are Dome Tubes; and #5 is Dome^2^. In addition to the damage from Li photon radiation, the escaped plasma core and scattered particles from the evolving Li secondary plasma also cause surfaces damage. Figure 4b presents the particle flux plotted as vectors in logarithmic scale to clearly show the location and direction of the disruption impact. The particle flux is very high at the 0.5 ms of the 1.0 ms disruption above the Baffle surface.

**Figure 5:**
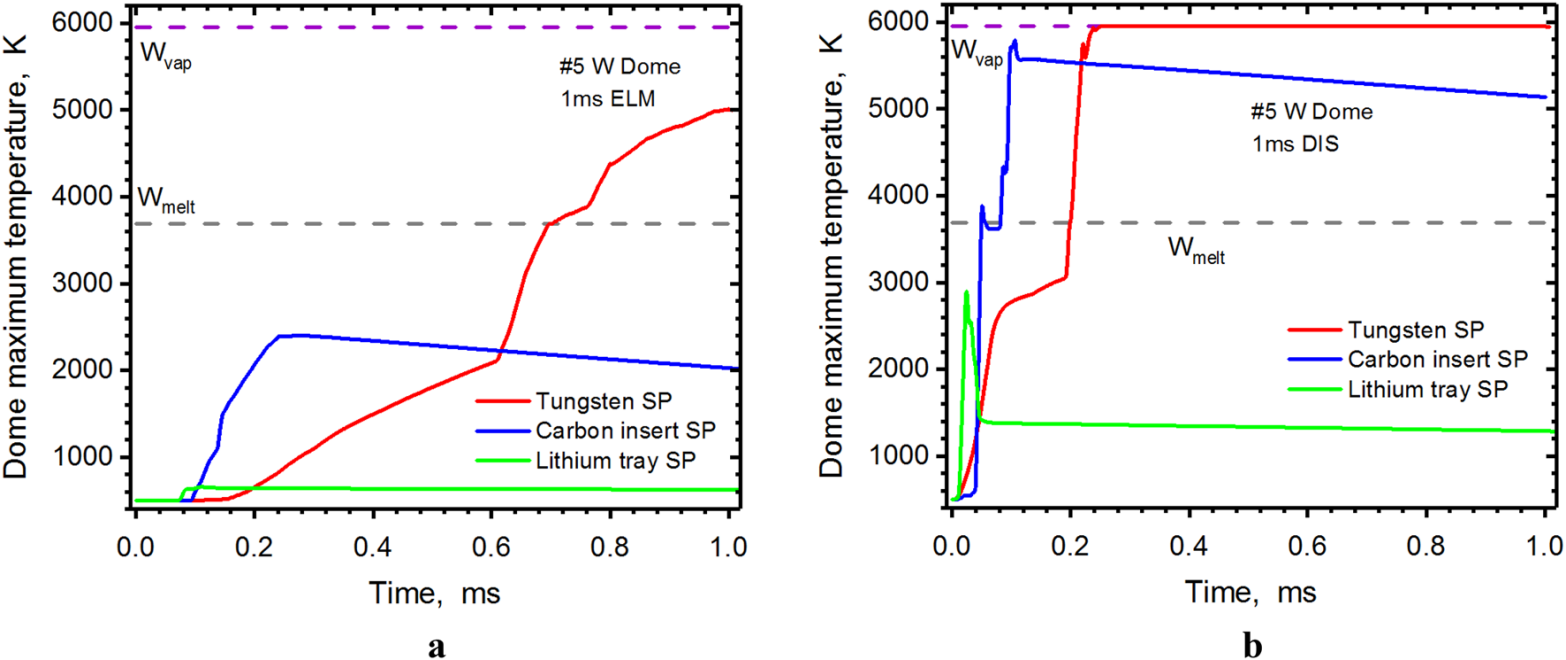
HEIGHTS simulation of PFC transient response: #5 Dome maximum surface temperature during 1.0 ms ELM (**a**, **b**) 1.0 ms disruption (**b**), see Fig. 4a for surfaces location. The images were prepared using OriginPro V2020.

**Figure 6:**
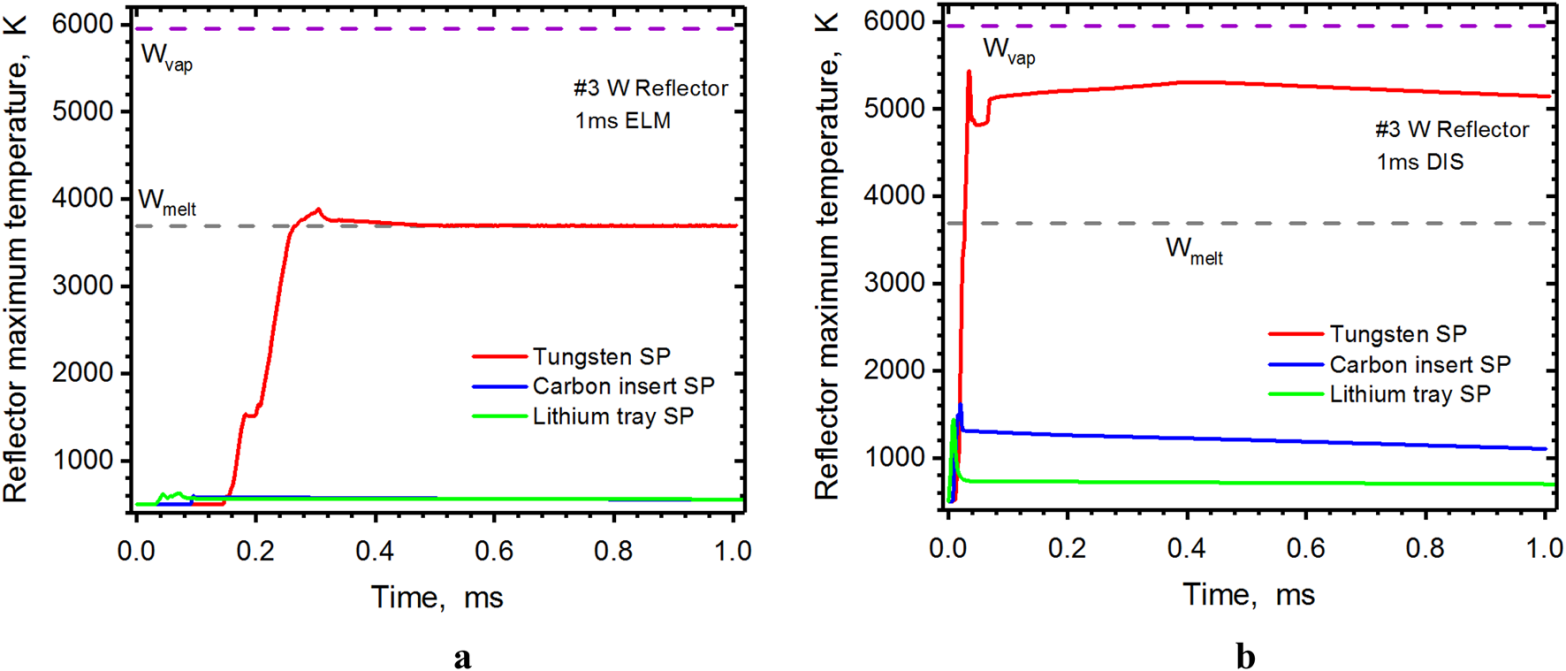
HEIGHTS simulation of PFC transient response: #3 Reflector maximum surface temperature during 1.0 ms ELM (**a**), 1.0 ms disruption (**b**), see Fig. 4a for surfaces location. The images were prepared using OriginPro V2020.

In our previous calculations, we found a critical damage spot on the #5 Dome surface for the full tungsten divertor during the disruption^2^. This unexpected spot will also be melted during the ELM event. Using a small carbon insert at the SP solves this problem for the ELM but during the disruption the Dome spot will still be melted. The use of the lithium trays or structure solves completely the overheating problem on the Dome surface (see Fig. 5). The green curve (Li case) shows the Dome surface temperatures during an ELM to be less than 800 K and less than 3000 K during a disruption event. The second overheating area we predicted for ITER-like design was the #3 Reflector. The Li secondary plasma cloud also greatly decreases the heat load on this surface. Figure 6 shows the great reduction in the Reflector surface temperature for the lithium case (green curve) during the disruption event.

**Figure 7:**
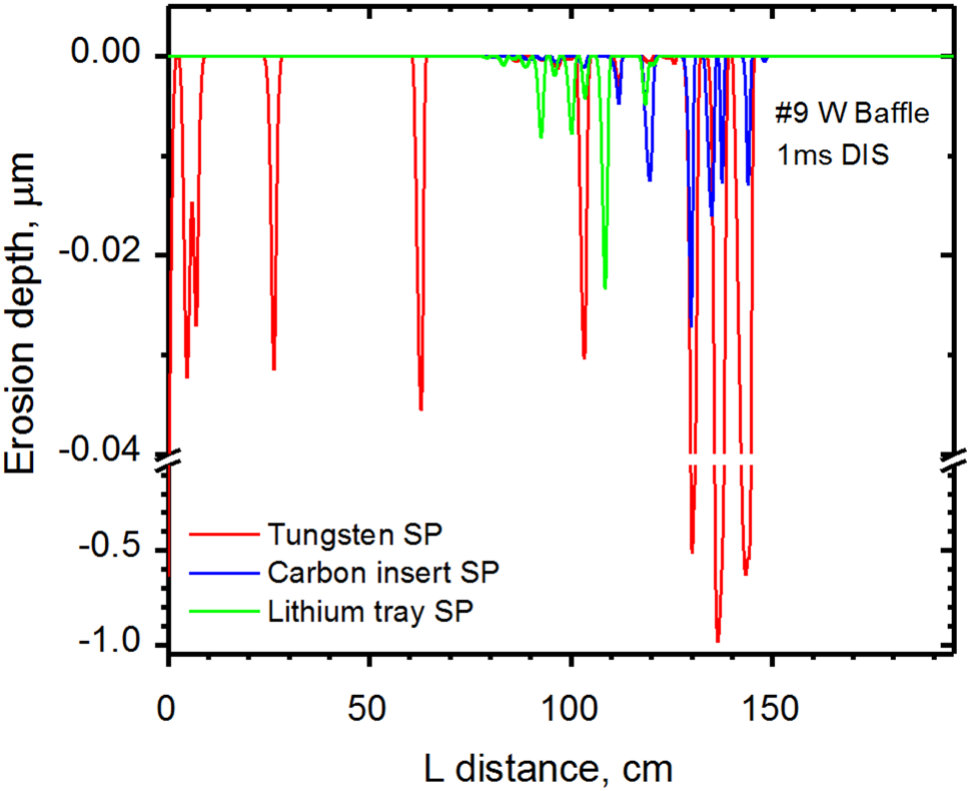
HEIGHTS simulation of the erosion depth of #9 Baffle surface during 1.0 ms disruption, see Fig. 4a for surfaces location. The images were prepared using OriginPro V2020.

**Figure 8:**
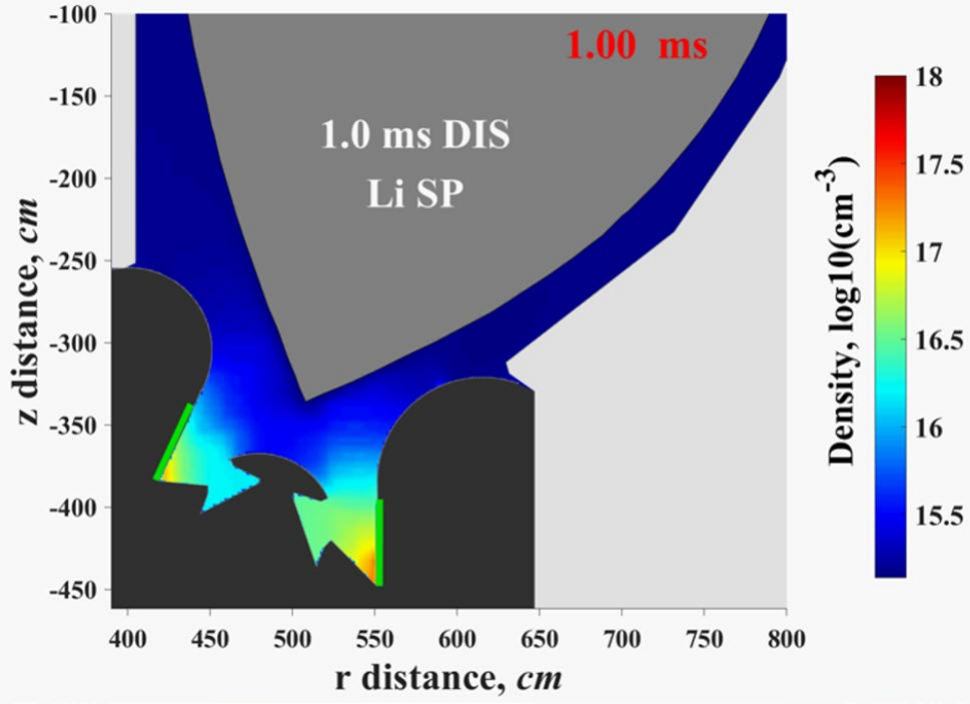
HEIGHTS calculated Li secondary plasma density in divertor space after the 1.0 ms disruption. (See Supplementary Video S2).

The predicted unexpected erosion locations during the disruption is on the outer #9 Baffle. Figure 7 shows the erosion shape of the Baffle surface after the 1.0 ms disruption. As shown in this plot, the maximum erosion depth for the full tungsten divertor case (red curve) can reach up to ~ 1 μm. The use of low-Z materials reduces erosion of up to ten times lower in crater depth at the end of the 1.0 ms disruption. We expect that the implementation of a full lithium coating of the DEMO divertor components will also mitigate this problem. The Li plasma density is insufficient above the Baffle surface (Fig. 8). (See Supplementary Video S2 for the HEIGHTS simulated dynamics of the Li secondary plasma initiation and expansion from the SP location along the divertor component surfaces). The #9 Baffle erosion is a result of insufficient secondary plasma shielding, i.e. insufficient Li cloud formation and expansion along the Baffle surface. The presence of the other lithium DEMO surfaces should boost the developed Li plasma shielding, mitigate erosion, and enhance component lifetime.

## Summary and conclusion

The main energy flux coming from the core plasma in magnetically confined fusion reactors into the divertor space is concentrated in a relatively narrow area around the separatrix, the border between the closed and open magnetic field line areas^17^. The success of the tokamak reactors is mainly determined by the best choice of materials for various components of the device. In the divertor space, a secondary plasma cloud originated from the divertor surface material is developed due to the hot hydrogen plasma interaction and deposition in divertor materials during plasma instabilities. Traditional materials such as tungsten, beryllium, or carbon initially appear to solve plasma-material interaction problems for ITER-like projects, albeit including procedure of divertor replacement after thousands of pulses. Each of these well-tested materials in the existing tokamaks has serious disadvantages along with certain advantages. As a result, several problems arise such as radiation cooling due to high-Z plasma impurity, high heat deposition, large erosion, fuel retention, accumulation of dust, etc. For plasma transient events as for the DEMO project where much higher divertor heat flux is expected, new material and design solutions are required. An obvious step towards reducing the high-Z core plasma cooling, mitigating erosion, reduce fuel retention, etc. is to change the material of the divertor plates to a low-Z replenishable material such as lithium, where several corresponding studies are currently being studied in NSTX-U, DIII-D, and EAST tokamaks^18–20^.

The objective of this work was to study in comprehensive integrated simulation the advantages of liquid lithium response during plasma transient events in ITER-like design and for future DEMO project performances using the original full exact 3D ITER design and parameters. For this purpose, we have enhanced our HEIGHTS full 3D integrated simulation package for lithium calculations including detail photon radiation transport and focused the present study to investigate heat loads and damages to various PFCs surfaces during the plasma transient events of ELMs and disruptions.

Our simulations predicted significant reduction in the heat loading and damage to the divertor nearby and internal components in the case when lithium is used on the divertor plates. When tungsten or carbon are used on the divertor plate, significant melting and vaporization spots (less for carbon than tungsten) can occur on the reflector, dome, and stainless steel tubes, and even parts of the first walls can melt due to the high radiation power of the secondary divertor plasma. Lithium photon radiation deposition into the divertor and nearby surfaces was significantly decreased by two orders of magnitude compared to tungsten and one order of magnitude compared to carbon. This analysis showed that using liquid lithium for ITER-like surfaces and future DEMO can lead to significant enhancement in components lifetime.

## Methods

**Figure 9:**
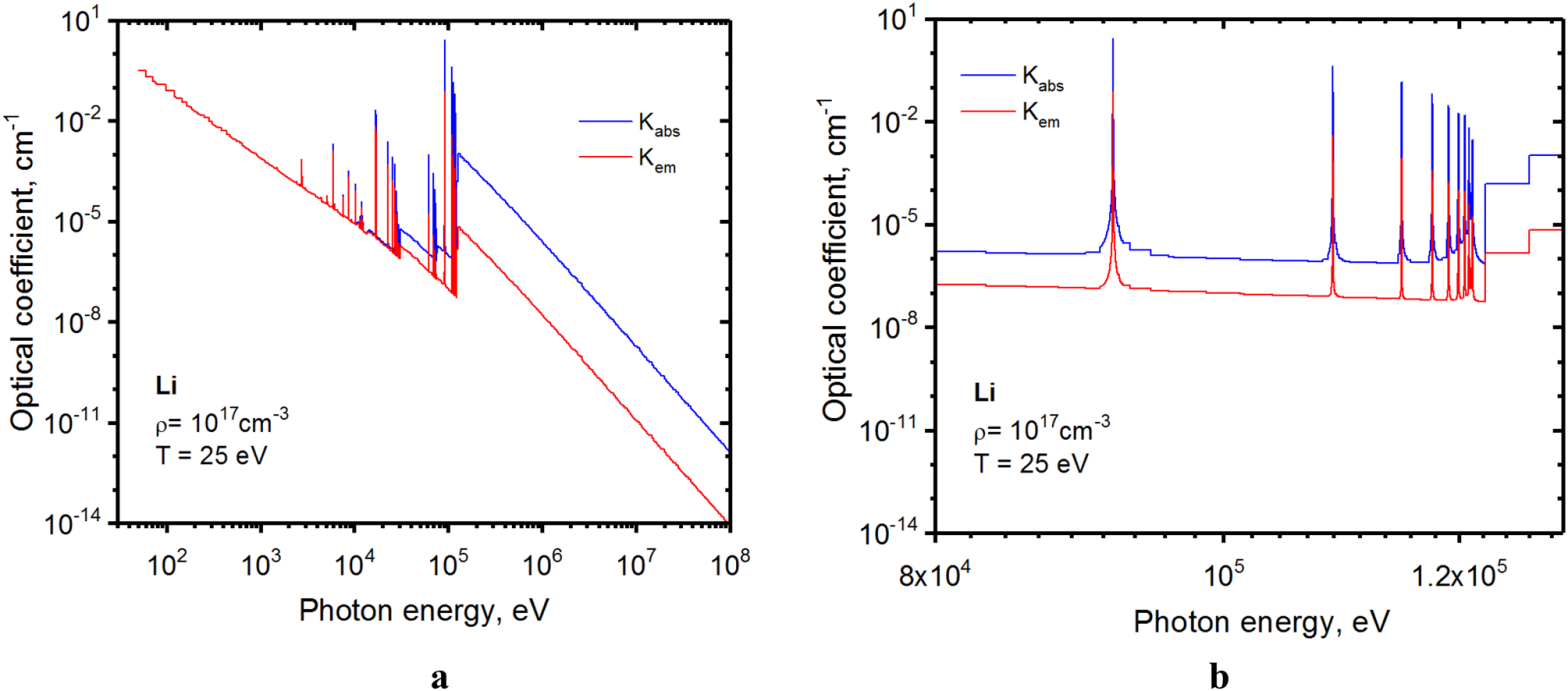
Optimized opacities of lithium plasma for RT calculations: full spectrum **a**, and fine structure **b**. The images were prepared using OriginPro V2020.

Methods details, including statements of data availability and any associated accession codes and references, are also available at https://doi.org/10.1038/s41598-021-81510-2 and https://doi.org/10.1038/s41598-022-08837-2. We upgraded our HEIGHTS radiation transport (RT) calculation in lithium plasma with a detailed consideration of energy transfer in strong lines along with the continuum spectra. To allow simulation of RT having many strong lines, we optimized the initial opacity tables and separated the full plasma spectrum into spectral groups where optical coefficients are relatively invariable. Using such technique, the opacity tables were reduced by an order of magnitude for complex elements as tungsten and by two orders of magnitude for the lighter elements such as carbon and lithium. Figure 9 shows an example of optimization of lithium opacities for 25 eV temperature and 10^17^ cm^-3^ ionic concentration. Because the plasma spectrum depends critically on the temperature, the collected spectral groups are created for the large set of temperatures. The spectrum fine structure with separation of strong lines in the area of photon energy ~ 10 keV is shown in Fig. 9b.

## Supplementary Information

Supplementary Information.

Supplementary Video 1.

Supplementary Video 2.

## Data Availability

The data that support the findings of this study are stored on Purdue Servers and on Argonne National Laboratory Bebop cluster and are available from the corresponding authors upon reasonable request.
