# Supplementary material for: Liquid lithium as divertor material to mitigate severe damage of nearby components during plasma transients
Source: Sci Rep. 2022 Nov 5;12:18782. doi: 10.1038/s41598-022-21866-1 (PMC9637163; doi:10.1038/s41598-022-21866-1)
Supplement: Supplementary file 1 — Supplementary Information. [file 41598_2022_21866_MOESM1_ESM.docx]

**Liquid lithium as divertor material to mitigate severe damage of nearby components during plasma transients**

V. Sizyuk* and A. Hassanein

Center for Materials under Extreme Environment (CMUXE)

Purdue University, West Lafayette, IN 47907, USA

^*^ **E-mail address:** [vsizyuk@purdue.edu](mailto:vsizyuk@purdue.edu)

**Description of Additional Supplementary Files:**

1. **File name:** Supplementary_Video_S1.mp4

**Description:** HEIGHTS simulated evolution of the core escaped particles in ITER SOL.

2. **File name:** Supplementary_Video_S2.mp4

**Description:** HEIGHTS calculated Li secondary plasma expansion during the 1.0 ms disruption.
